# A 515,579-Genome Reference Panel Improves Rare-Variant Imputation Across Multiple Underrepresented Populations

**DOI:** 10.64898/2026.08.25.26361247

**Published:** 2026-08-31

**Authors:** Franjo Ivankovic, Arthur Ko, M. Morgan Aster, Mary K Balaconis, Eric Banks, Matthew Bemis, Kristian R Cibulskis, Kylee Degatano, Laura D Gauthier, George Grant, Aaron Hatcher, Christopher Kachulis, Konrad J Karczewski, Sofia M Labrecque, Jonathan Lawson, Calwing Liao, Ricky Magner, Ruchi Munshi, Michael C Schatz, Patrick M Schultz, Saloni P Shah, Elizabeth A Sheets, Kathleen Tibbetts, Kyle A Vernest, Robert Ye, Stacey Gabriel, Niall J Lennon, Benjamin M Neale, Brian L Browning, Lee T Lichtenstein

## Abstract

Genotype imputation remains essential for large-scale human genetics studies, but its performance is limited by the size and ancestral diversity of available reference panels, reducing accuracy for rare variants and underrepresented populations. Here, we present a cloud-based imputation service built on a multi-ancestry reference panel derived from 515,579 jointly phased genomes from the *All of Us* (N=414,830) and National Human Genome Research Institute’s *Analysis, Visualization, and Informatics Lab-space* (AnVIL, N=100,749) datasets. The *All of Us +* AnVIL reference panel is highly diverse and includes 261,163 participants most genetically similar to non-European reference populations, spanning 665,398,839 high-quality autosomal sites, representing a nearly 50% increase over TOPMed, the previous largest imputation service. Across multiple ancestry groups, the panel enables accurate imputation (empirical R² > 0.8) for variants with allele frequencies as low as 0.2%, extending reliable imputation into the rare-variant frequency spectrum, including allele frequencies down to 0.002% and 0.006% for samples with European ancestry and African ancestry in the United States, respectively. Compared with TOPMed, the panel improves imputation accuracy across all ancestry groups except Africans, and recovers additional trait-associated variants not represented in existing reference panels. To facilitate broad community access while preserving participant privacy, we deploy the panel through a secure cloud-based imputation platform using privacy-preserving recombined haplotypes. This resource establishes a new foundation for genome-wide association studies (GWAS) and fine-mapping, especially in previously underrepresented populations.

## Full Text Availability

The license terms selected by the author(s) for this preprint version do not permit archiving in PMC. The full text is available from the preprint server.

